# The Validity and Reliability of the Persian Version of the Family Health Climate Scale (FHC-Scale) in Female Students and Their Mothers in Iran 2019

**DOI:** 10.1155/2021/8845716

**Published:** 2021-02-05

**Authors:** Jeyran Ostovarfar, Mohammad Hossein Kaveh, Leila Ghahramani, Masoud Karimi, Abdolrahim Asadollahi, Razie Zare

**Affiliations:** ^1^Department of Health Promotion, Shiraz University of Medical Sciences School of Health and Nutrition, Shiraz 71557, Iran; ^2^Department of Health Promotion, School of Health, Shiraz University of Medical Sciences, Shiraz 71557, Iran; ^3^Industrial & Organizational Psychology Department, Faculty Education & Psychology, Shahid Chamran University, Ahvaz 61336, Iran

## Abstract

**Objectives:**

This study examined the validity and reliability of the Family Health Climate Scale (FHC-Scale) among Iranian families. Second, can it be attributed to other family members by measuring the health climate in one person?

**Method:**

In total, 261 female students and 196 mothers completed the FHC-Scale. The study instrument was a Persian version of the FHC-Scale prepared through a translation and back-translation process.

**Results:**

The results showed that the Persian version of the FHC-Scale is acceptable. Cronbach's alpha coefficient for FHC-PA in female students and their mothers, respectively, was 0.88 and 0.86 for the whole scale. Cronbach's alpha coefficient for FHC-NU in female students and their mothers, respectively, was 0.83 and 0.92 for the whole scale. The CVI values for all the items were equal to or above 0.8, and the CVR value for the total scale was 0.90.

**Conclusion:**

The Persian version of the FHC-Scale is therefore an effective tool for evaluating the different dimensions of family health climate in the Iranian population.

## 1. Introduction

Health behaviors learned during childhood easily remain in people's lifestyles and will have more permanent effects compared to behaviors obtained in adulthood [1]. A healthy lifestyle is determined by many factors, including individual, social, physical, and environmental factors and their interaction. People's health behavior patterns are not developed and maintained in a vacuum. Health behaviors are embedded in social contexts and are influenced by social relationships, which are among the most important social contexts of the family [2, 3].

### 1.1. Health Behaviors in the Family Environment

Many studies have focused on the effects of the family environment on the parent-child relationship and the effect of parental behaviors on children's behaviors. Previous research has shown the important role of parents in creating a healthy lifestyle in their children [4, 5]. Parents are the gatekeepers who determine the healthy nutrition and physical activity of their children. For example, by determining what food is available at home, they shape their children's eating habits. Access to sports equipment is also provided by parents. Other influential factors include parenting styles, such as role modeling, supervision, support, and encouragement [6–9], and more general concepts such as parenting styles [10, 11]. In fact, parents' role modeling of healthy behaviors can increase healthy behaviors in their children [12]. In general, positive parenting is positively correlated to children's healthy eating behaviors and physical activity levels [13, 14]. Family members, on the other hand, interact in a shared family environment. Individuals and the interaction between them affect the family environment, which in turn affects individuals and their interactions. Therefore, the “family level” is beyond individuals and their shared environment, and consequently, the family can be considered a system [15].

### 1.2. Family Health Climate in the Family System

Evidence has shown a link between diet and physical activity [16, 17]. Family refers to a group of individuals, and all members of this group have specific motivations, influences, and behaviors. Individuals within a family interact with each other. These interactions occur over a long period of time and at a high frequency, forming the “family system” that is one of the fundamental components of the family environment. Approaching family as a system, the fundamental questions are how the family environment affects an individual's health behaviors and how this effect can be described. It has been stated that a particular aspect of family members' interactions shapes individuals' physical activity and eating behaviors, which is called the “climate.” In description of the environmental dimensions of the family that lead to the creation of individual health behaviors, the term climate has been judged as a special feature [18, 19]. The term “family health climate” was introduced by Niermann et al. [20].

### 1.3. Health-Related Behaviors and Family Health Climate

Family health climate has been defined as the shared perceptions and cognitions concerning health and health behaviors. It reflects the individual experience of daily family life, evaluation of health-related topics, and expectations with respect to typical values, behavior routines, and interaction patterns within the family [20].

Parents and children are parts of the family and influence each other's behaviors, and this indicates reciprocal influence. These family interactions and perspectives are a part of the family health climate [18, 19]. Because a specific understanding of the family climate predicts particular behaviors [21], and behaviors such as eating and physical activity are completely different in spite of being related to health [22]; this tool includes climate perceptions that refer to these behaviors [20].

According to a scale developed by Niermann et al., the family health climate influences the health behaviors of family members. Family health climate can be measured using the Family Health Climate for Physical Activity (FHC-PA) Scale and the Family Health Climate for Nutrition (FHC-NU) Scale. By measuring the family health climate of a family member, the results can be attributed to the whole family [20]. To prove this claim, the present study was conducted on two family members.

The Family Health Climate Scale focuses on the typical aspects of the family environment (e.g., communication, time together, encouragement, and connectedness) and represents emotional, cognitive, and instrumental aspects of the family environment. According to the specificity of different health behaviors, this instrument includes two separate scales: FHC-PA with 14 questions and FHC-NU with 17 questions [20].

### 1.4. Objects

In response to the lack of investigations of the validity and reliability of the Family Health Climate Scale after its development and validation by Niermann et al., the present study is aimed at examining the validity and reliability of this questionnaire among Iranian families. It is also aimed at determining whether the measurement of health climate in one person can be attributed to other family members.

## 2. Materials and Methods

This cross-sectional study was conducted to evaluate the validity and reliability of the Family Health Climate Scale and to assess its generalizability from an individual to other family members among Iranian female students and their mothers in Shiraz 2019.

### 2.1. Participants

The study participants were fifth-grade female students who studied in Shiraz schools as the first target group. Since various studies have indicated that mothers' healthy behaviors have a greater impact on children compared to the paternal model [23], emotional dependence is stronger in the mother-daughter relationship and the nature of the relationship plays an important role in girls' social and psychological well-being [24], and the mother-daughter relationship is unique and significant because of the solidarity and support provided by women [25, 26], mothers were selected as the second group.

The participants were selected from the four educational districts in Shiraz using cluster sampling. At first, two out of the four districts were randomly selected (districts 2 and 4). In each of the selected districts, four public schools were randomly selected. In each school, two classes were randomly selected, and eventually, 261 female students were selected. After the students were explained about the study objectives, they were given two questionnaires: one for themselves and one for their mothers. The students were asked to give the questionnaires to their mothers. The students and their mothers were required to complete the questionnaires and return them to the researcher within one week. Because of the researcher's relationship with the students, all students completed the questionnaires, and 196 mothers completed the questionnaires that are about 75%.

### 2.2. Family Health Climate Scale

Family Health Climate Scale includes two separate scales: FHC-PA and FHC-NU. FHC-PA contains 14 questions with three subscales (*value*, for example, “it is normal in our family to be physically active in our leisure time”; *cohesion*, for example, “…we have fun doing physical activities together (e.g., bike tours and hikes)”; and *information*, for example, “we collect information (e.g., on the internet) on physical activity and exercise”) [20]. FHC-NU consists of 17 questions with four subscales (*value*, for example, “a healthy diet plays an important role in our lives”; *communication*, for example, “we talk about which foods are healthy”; *cohesion*, for example, “we appreciate spending time together during meals”; and *consensus*, for example, “we rarely argue about food- or diet-related matters”). All questions were begun with “In our family…,” and answers were given on a four-point rating scale (0 = “definitely false,” 1 = “rather false,” 2 = “rather true,” and 3 = “definitely true”) [20].

In a study by Niermann et al., mothers, fathers, and adolescents completed a questionnaire separately. The internal correlation was *α*FHC‐PA = 0.92 and *α*FHC‐NU = 0.86 for the mothers, *α*FHC‐PA = 0.90 and *α*FHC‐NU = 0.86 for the fathers, and *α*FHC‐PA = 0.90 and *α*FHC‐NU = 0.85 for the adolescents [20].

### 2.3. Persian Translation of the Family Health Climate Scale

The Family Health Climate Scale was translated according to the four sequential stages of translation and back-translation as recommended by Chen et al. [27]. The translation instructions emphasized conceptual rather than literal accuracy as well as the need to use an acceptable linguistic approach for the majority of Persian-speaking participants. This meant an avoidance of technical terms and jargons.

The bilingual expert panel consisted of the original translator, experts in public health, and experts in translation and development of questionnaires. This panel was required to sort out discrepancies and to reach a consensus regarding the translated version of the scale.

The initial forward translation into Persian was back-translated to English by a single independent translator for whom English was the mother tongue and who had no knowledge related to the questionnaire. The back-translated English version was then cross-matched with the original English version.

A pretesting phase was carried out on ten participants from the same sampling group who were not involved in the main study. The instrument was administered to the participants after a short explanation of the content. Face-to-face interview sessions were conducted by the primary investigator. The answers obtained from these sessions were matched with the actual responses given by the respondents. The respondents were also interviewed regarding the questionnaire content and ease of understanding.

## 3. Results

### 3.1. Content Validity

The face and content validity of the translated version was assessed by a group of experts consisting of 15 academic staff of Shiraz University of Medical Sciences who had work experience in the field of Epidemiology and Health Education and Promotion. Clarity of the wording, placement of the items, and scoring were checked by the experts at this stage, and their feedbacks were followed to make corrections. Content Validity Ratio (CVR) and Content Validity Index (CVI) were calculated based on the experts' opinions in terms of relevance, clarity, and simplicity of the items in the translated scale. The CVI values were equal to or above 0.8 for all items, and the CVR value was 0.90 for the total scale. In the end, to confirm face validity, the scale was given to 10 female students and their mothers for feedback on the simplicity of the scale and understandability of items so that possible ambiguities could be further refined.

### 3.2. Construct Validity

In the first step, the construct validity of the Family Health Climate Scale was determined to extract the number of hidden factors using exploratory factor analysis. For both FHC-PA and FHC-NU scales, assumptions for exploratory factor analysis were confirmed (FHC-PA: Kaiser-Meyer-Olkin = 0.84, Bartlett's test of sphericity = 2522.66, df = 91, *p* < 0.001; FHC-NU: Kaiser-Meyer-Olkin = 0.82, Bartlett's test of sphericity = 1449.02, df = 136, *p* < 0.001). The results of these indicators showed an optimal correlation between the variables that made factor analysis possible. Factors in the test were extracted by principal component analysis and varimax rotation for both FHC-PA and FHC-NU scales. In this model, three factors were extracted for FHC-PA according to eigenvalues higher than one. The eigenvalues of the three hidden factors were 5.76, 1.84, and 1.62 after rotation. The three extracted factors explained 65.91% of the total variance of the construction of the FHC-PA in female students. Considering FHC-NU, four factors were extracted according to eigenvalues higher than one. The eigenvalues of the four hidden factors were 4.74, 2.54, 1.51, and 1.12 after rotation. The four extracted factors explained 58.42% of the total variance of the construction of FHC-NU in female students. The exploratory factors extracted from the Family Health Climate Scale are presented in Table 1.

**Figure 1:**
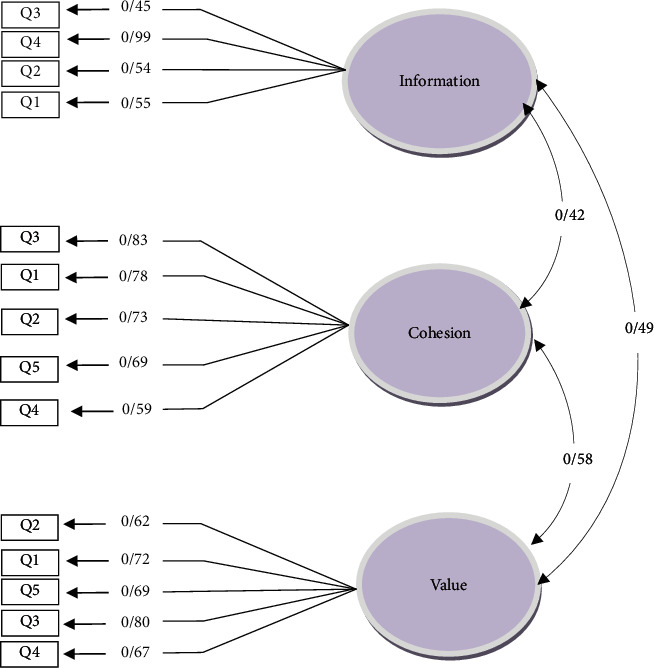
Factor structure of FHC-PA (mothers' sample).

**Figure 2:**
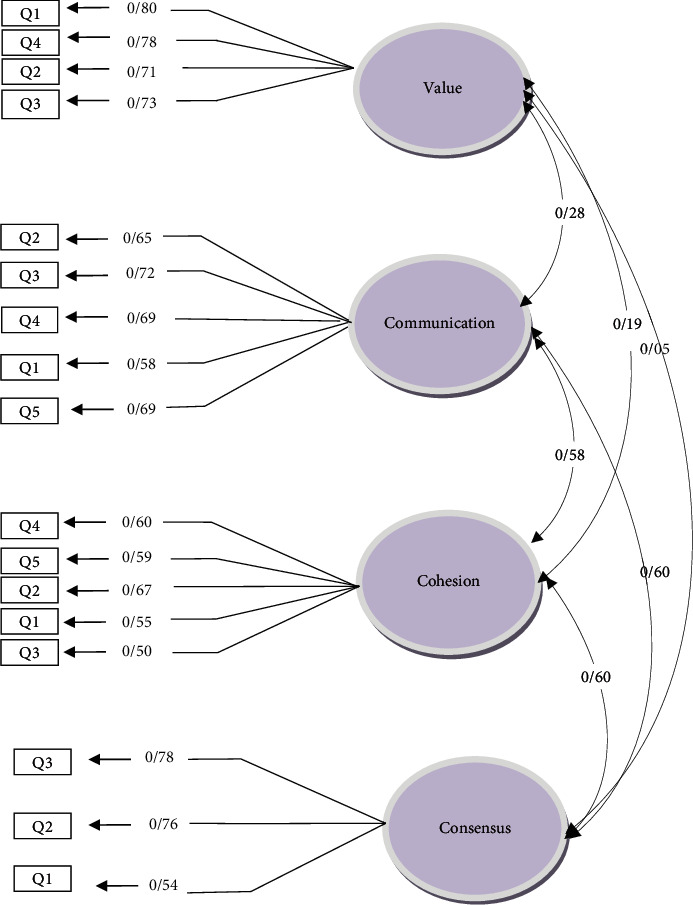
Factor structure of FHC-NU (mothers' sample).

In the second step, to assess the fitness of the final model of the three-factor structure of FHC-PA and the four-factor structure of FHC-NU, confirmatory factor analysis was performed on the mothers. To test the assumed factor structures (confirmation of the extracted factors), confirmatory factor analysis was performed using AMOS software. To this end, a model based on the previous information about data structure was developed, and the data for the model were analyzed. Findings from the implementation of confirmatory factor analysis through eight evaluation criteria, including the value of the chi-square index, normed *χ*^2^ measure index (the chi-square ratio of the degree of freedom), goodness of fit index (GFI), adjusted goodness of fit index (AGFI), normed fit index (NFI), comparative fit index (CFI), incremental fit index (IFI), Tucker-Lewis index (TLI), and root mean square error of approximation (RMSEA), are shown in Table 2. The factor structure of the FHC-PA scale in the present study is presented in Figure 1. Accordingly, all items had moderate to high factor loads (*p* < 0.001). Additionally, the indicators of the confirmatory factor analysis model of FHC-PA demonstrated that the measures of the indicators were close to the fitness criteria and that the confirmatory factor analysis model had an acceptable fit. The fitness indicators of the factor analysis model of FHC-PA are presented in Table 2. As the table depicts, the fitness indices of the confirmatory factor analysis model of FHC-PA in the present study were acceptable. The factor structure of FHC-NU is shown in Figure 2. Accordingly, all items had moderate to high factor loads (*p* < 0.001). Moreover, the indicators of the confirmatory factor analysis model of FHC-NU revealed that the measures of the indicators were close to the fitness criteria and that the confirmatory factor analysis model had an acceptable fit. The fitness indicators of the factor analysis model of FHC-NU are presented in Table 3. As the table depicts, the fitness indices of the confirmatory factor analysis model of FHC-NU in the present study were acceptable.

#### 3.2.1. Reliability

To evaluate the reliability of both FHC-PA and FHC-NU scales, Cronbach's alpha and split-half methods were used; the results of which are reported in Table 4. Accordingly, both FHC-PA and FHC-NU scales had good reliability.

### 3.3. Perception of Mothers and Female Students of the Family Health Climate

Paired *t*-test was used to analyze different perceptions of family health climate among students and their mothers. The results of paired sample *t*-test are presented in Table 5. The results revealed a significant difference between the students' and their mothers' perceptions of FHC-NU.

#### 3.3.1. Components

The results of paired *t*-test for FHC-NU components showed that the communication component was perceived differently by the students and their parents (*p* < 0.01). Accordingly, the mean score of communication perceived by the students was significantly higher compared to that by the mothers.

Based on Table 6, the results of paired sample *t*-test indicated a significant difference between the students and their parents regarding their perceptions of FHC-PA.

#### 3.3.2. Components

The results of paired *t*-test for FHC-PA components showed that the value, cohesion, and to some extent information components were perceived differently by the students and their parents (*p* < 0.05). Accordingly, the mean scores of value and cohesion perceived by the students were significantly higher compared to those by the mothers.

## 4. Discussion

This was the first study on the validity and reliability of the Family Health Climate Scale after its development and validation by Niermann et al. This study confirmed that the Family Health Climate Scale had appropriate construct validity, content validity, discriminant validity, and internal consistency, which indicated that the Persian version of the scale followed a logical and appropriate trend.

Reliability means repeatability, and it is assessed through various methods [28]. In the present study, reliability was evaluated using internal consistency and split-half methods. Cronbach's alpha coefficient for FHC-PA was 0.88 in the female students and 0.86 in the mothers. In the research carried out by Niermann et al., the internal consistency coefficients ranged from 0.81 to 0.9 for FHC-PA [20]. In the current study, Cronbach's alpha coefficient for FHC-NU was 0.84 among the female students and 0.92 among the mothers. In the study by Niermann et al., the internal consistency coefficients ranged from 0.74 to 0.90 for FHC-NU [20].

The reliability coefficient obtained using the split-half method for FHC-PA was 0.73 among the female students and 0.70 in their mothers. Considering FHC-NU, the reliability coefficient was 0.71 for the female students and 0.84 for their mothers. According to McKelvie [29], these values indicated the acceptable reliability of the Persian version of the scale.

The fitness indicators of the confirmatory factor analysis model presented in Tables 3 and 4 showed that both FHC-PA and FHC-NU components of the Persian version of the Family Health Climate Scale had desirable conditions and were consistent with the fitness indicators of the confirmatory factor analysis model of the original version created by Niermann et al. The validity of the scale was also acceptable according to the commonly recommended fit indices. In this study, *χ*^2^/df, CFI, standardized root mean square residual (SRMR), and RMSEA were used to assess the goodness of fit. A good fit is indicated by 0 ≤ *χ*^2^/df ≤ 2, 0.97 ≤ CFI ≤ 1, 0 ≤ SRMR ≤ 0.05, and RMSEA ≤ 0.05, while values 2 < *χ*^2^/df ≤ 3, 0.95 ≤ CF < 0.97, 0.05 < SRMR ≤ 0.10, and 0.05 < RMSEA ≤ 0.08 indicate an acceptable fit [30]. The concepts of nutrition and physical activity included in the Family Health Climate Scale are international concepts that are compatible with different cultures and languages and seem to be applicable all over the world; therefore, the consistency of the results of the present study with the results of the study of Niermann et al. is reasonable.

According to the results presented in Table 5, the mean score of FHC-NU was significantly higher in female students than in their mothers. The mean score of the communication subscale in FHC-NU was also significantly higher among the female students compared to their mothers. Mothers generally use role modeling to communicate health behaviors to their children to control their eating behaviors and constantly remind their children about healthy eating [31, 32]. As a result, children feel that talking about and supporting healthy eating is one of the family's priorities. Therefore, they understood the family health climate more than their mothers did.

In the current study, the mean scores of almost all subscales and the total mean score of the FHC-PA scale were higher among the female students in comparison to their mothers. This can be justified by the fact that the Family Health Climate Scale measures the family level, is scored by individuals, and is associated with individuals' understandings of the motivational and behavioral aspects of other family members [20] and that parents, especially mothers, use motivational messages and try to provide opportunities to engage their children in physical activities [33].

## 5. Limitations

Family health climate was measured only among females, because females care more about their health compared to males [34, 35]. Thus, a more comprehensive study in which a statistical population of males is also present is recommended to be conducted on the issue. The other limitation of this research was the inability to have access to the target group to retest the Family Health Climate Scale due to the prevalence of the Coronavirus Disease (COVID-19).

## 6. Conclusion

The family health climate describes a family level, which is suspected to affect the health behaviors of the family members [20]. The Family Health Climate Scale is a valid and reliable instrument to measure the climate about healthy eating and physical activity in the family context.

Even in the same family, people seem to have different perceptions of the family health climate, which depends on the type of behaviors and motivations that other family members give to the person about healthy behaviors. This hypothesis can be tested by measuring the family health climate in other family members. In the present study, the high scores of the female students in both FHC-NU and FHC-PA scales compared to their mothers indicated that the mothers, as role models, tried to create a positive health climate for their children so as to encourage them towards healthy eating and physical activity.

Overall, the results of the present study showed that the Family Health Climate Scale could be used effectively in cross-cultural comparative studies among Persian-speaking communities.

## Figures and Tables

**Table 1:** Exploratory factors extracted from the Family Health Climate Scale (student sample).

Scales	Domain	Original item code	Factor loadings	Item share	Eigenvalues
FHC-PA	Information	Q3	0.846	0.982	5.57
		Q4	0.863	0.982	
		Q1	0.786	0.503	
		Q2	0.734	0.506	
	Cohesion	Q3	0.841	0.630	1.84
		Q1	0.840	0.578	
		Q2	0.769	0.508	
		Q5	0.640	0.511	
		Q4	0.552	0.411	
	Value	Q2	0.777	0.431	1.62
		Q1	0.772	0.486	
		Q5	0.742	0.448	
		Q3	0.701	0.546	
		Q4	0.657	0.418	
FHC-NU	Communication	Q2	0.797	0.436	4.74
		Q3	0.740	0.433	
		Q4	0.703	0.436	
		Q1	0.653	0.653	
		Q5	0.580	0.450	
	Value	Q1	0.849	0.551	2.54
		Q4	0.817	0.516	
		Q2	0.810	0.451	
		Q3	0.786	0.473	
	Cohesion	Q4	0.819	0.361	1.51
		Q5	0.686	0.355	
		Q2	0.586	0.417	
		Q1	0.564	0.331	
		Q3	0.554	0.275	
	Consensus	Q3	0.786	0.450	1.12
		Q2	0.766	0.430	
		Q1	0.618	0.205	

**Table 2:** The indicators of fitness of the factor analysis of the FHC-PA scale among the mothers.

Structure fitness indicators	*χ* ^2^	df	*χ* ^2^/df	GFI	AGFI	IFI	TLI	CFI	NFI	RMSEA
Three-dimensional structure	222.703	73	3.051	0.88	0.83	0.94	0.92	0.94	0.91	0.09

GFI: goodness of fit index; AGFI: adjusted goodness of fit index; IFI: incremental fit index; TLI: Tucker-Lewis fit index; CFI: comparative fit index; NFI: normed fit index; RMSEA: root mean square error of approximation.

**Table 3:** The indicators of fitness of the factor analysis of the FHC-NU scale in the mothers.

Structure fitness indicators	*χ* ^2^	df	*χ* ^2^/df	GFI	AGFI	IFI	TLI	CFI	NFI	RMSEA
Four-dimensional structure	205.802	113	1.821	0.91	0.93	0.91	0.92	0.93	0.86	0.05

GFI: goodness of fit index; AGFI: adjusted goodness of fit index; IFI: incremental fit index; TLI: Tucker-Lewis fit index; CFI: comparative fit index; NFI: normed fit index; RMSEA: root mean square error of approximation.

**Table 4:** The reliability coefficients of the Family Health Climate Scale.

Scales	Domains	Mothers				Female students			
		Cronbach's *α*		Split-half		Cronbach's *α*		Split-half	
FHC-PA	Information	0.84	0.86	0.70	0.70	0.87	0.88	0.75	0.73
	Cohesion	0.77		0.67		0.84		0.71	
	Value	0.84		0.78		0.82		0.75	
FHC-NU	Cohesion	0.96	0.92	0.87	0.84	0.79	0.83	0.72	0.71
	Value	0.92		0.88		0.84		0.82	
	Communication	0.97		0.93		0.71		0.69	
	Consensus	0.96		0.84		0.68		0.70	

**Table 5:** The results of paired sample *t*-test for comparison of FHC-NU as perceived by the female students and as rated by the mothers.

Variable	Mean	*N*	Std. deviation	Std. error of mean	Mean difference	*T*	df	Sig.
FHC-NU (female students)	28.48	196	9.837	0.702	2.045	2.235	195	0.027
FHC-NU (mothers)	26.43	196	8.999	0.642				
FHC-NU value (female students)	6.55	196	3.483	0.248	-2.295	-0.770	195	0.442
FHC-NU value (mothers)	6.78	196	2.467	0.176				
FHC-NU communication (female students)	10.83	196	3.837	0.274	1.984	5.434	195	0.000
FHC-NU communication (mothers)	8.84	196	3.715	0.265				
FHC-NU cohesion (female students)	7.69	196	4.356	0.311	0.729	1.887	195	0.061
FHC-NU cohesion (mothers)	6.96	196	3.858	0.275				
FHC-NU-consensus (female students)	3.40	196	2.936	0.209	-0.438	-1.576	195	0.117
FHC-NU consensus (mothers)	3.84	196	2.460	0.175				

**Table 6:** The results of paired sample *t*-test for comparison of FHC-PA as perceived by the student and as rated by the mothers.

Variable	Mean	*N*	Std. deviation	Std. error of mean	Mean difference	*T*	df	Sig.
FHC-PA (female student)	23.23	196	11.274	0.805	3.653	3.564	195	0.000
FHC-PA (mothers)	19.58	196	8.895	0.635				
FHC-PA value (female students)	8.88	196	4.904	0.350	1.469	3.321	195	0.001
FHC-PA value (mothers)	7.41	196	4.083	0.291				
FHC-PA information (female student)	5.35	196	4.480	0.320	0.760	1.849	195	0.066
FHC-PA information (mothers)	4.59	196	3.426	0.244				
FHC-PA cohesion (female student)	9.00	196	4.989	0.356	1.423	3.073	195	0.002
FHC-PA cohesion (mothers)	7.57	196	3.874	0.276				

## Data Availability

The data used to support the findings of this study are available from the corresponding author upon request.
